# Application of 3D reconstruction combined with 3D printing and CBL + SP in teaching gynecologic malignant tumors

**DOI:** 10.3389/fmed.2026.1915577

**Published:** 2026-09-14

**Authors:** Yaoxiang Zhong, Haikun Yang, Tingting Liu, Lvyang Li

**Affiliations:** Department of Gynaecology II, Meizhou People’s Hospital/Meizhou Academy of Medical Sciences, Meizhou, Guangdong, China

**Keywords:** 3D printing, case-based learning, gynecologic malignant tumors, medical education, standardized patient

## Abstract

**Objective:**

The synergic effect of integrating diversified teaching methods [3D visualization technology [3D reconstruction and printing], case-based learning [CBL]. and standardized patients [SPs]] in the clinical teaching of gynecologic tumors was determined.

**Methods:**

A case-control study design was proceeded in our hospital between September 2023 and March 2025 for clinical undergraduate interns and training physicians. They were divided into two groups: CBL + SP teaching mode (*n* = 54); and CBL + SP + 3D teaching mode (*n* = 54). A theoretical exam and a self-designed subjective questionnaire survey were used for the quantitative evaluation of teaching effectiveness.

**Results:**

The average score of the CBL + SP + 3D group in the theoretical exam was significantly higher than the CBL + SP group (*P* < 0.001). Students in the CBL + SP + 3D group performed significantly better than students in the CBL + SP group (*P* < 0.001)in the subjective grading: learning interest, classroom participation, self-learning capacity, comprehensive analytic capacity, clinical thinking capacity, effectiveness of visual teaching aids, and simulation of clinical consultation scenarios constructed using SP. The total score of satisfactions in the CBL + SP + 3D group was significantly higher than that in the CBL + SP group (*P* < 0.001). The total scores by the teachers on the students' performance in the CBL + SP + 3D group was significantly higher than that in the CBL + SP group.

**Conclusion:**

The CBL + SP + 3D model established a teaching system with integration of “virtualization + material object + interaction,” which enabled significant improvement in the learning interest of students and enhanced student stereoscopic cognition of the complex anatomy of gynecologic tumors and the pathologic structures, and deepened comprehensive thinking and clinical case analysis capacity.

## 1 Background

Gynecologic oncology, a medical specialty that involves the diagnosis and treatment of women with cervical, ovarian, and endometrial cancer, all of which have high incidence and mortality rates globally (1). Collectively, gynecological cancers accounted for 1,473,427 new cases and 680,372 deaths globally in 2022, with an incidence rate of 30.3 per 100,000 and a mortality rate of 13.2 per 100,000 (2). The complex anatomic structure and diverse pathologic features of malignant gynecologic tumors pose a huge challenge in clinical teaching. Teaching involving gynecologic tumors relies on a traditional mode in most medical institutions by integrating 2D imaging data with classroom instruction. However, abstract image information and lack of stereo perception are often challenging for students in understanding spatial relationships, lesion development, clinical reasoning, and surgical strategy design (3). Case-based learning (CBL) (4) and standardized patients (SPs) (5) have been widely adopted in medical teaching in recent years. These two methods simulate real clinical scenarios and help the clinical physicians improve problem-solving ability and develop humanistic communication skills. However, the current teaching mode is far from sufficient to effectively provide support for students to intuitively grasp the anatomic details and lesion evolution process due to the complexity of the spatial structure in gynecologic malignant tumors.

The development of a 3D visualization technique has offered the possibility of breaking through the bottlenecks of teaching and 3D reconstruction enables the transformation of traditional imaging data to a digital model, which clearly and visually presents the relationship between lesion morphology and adjacent structures (6). 3D printing further turns the digital model into material objects, increasing tactile cognition and spatial understanding for students (7, 8). 3D reconstruction and printing techniques have achieved active results in medical teaching, including the Departments of Orthopedics (7, 9), Respiratory Medicine (8), and Cardiovascular Medicine (6). 3D reconstruction and printing techniques also have active roles in the Department of Obstetrics and Gynecology Surgery or in clinical obstetrics and gynecology treatment, which enables clinical physicians to improve the efficiency and security of treatment (10–12).

However, the systemic integration and teaching mode development of 3D reconstruction 3D printing techniques in gynecologic oncology teaching are limited. Hence, in this prospective case-control study we combined the 3D reconstruction and the printing techniques into a teaching framework (CBL + SP) and established a teaching system with multimodal fusion involving theory-situation-material object. This study aimed to compare the teaching effect between the “CBL + SP + 3D” and “CBL + SP” teaching mode in areas, such as an improvement in spatial cognition, clinical thinking, and operative skills of students to compensate for the deficiency in clinical thinking training and the cultivation of spatial cognitive ability and improved teaching quality, as well as the practical cognitive ability of students.

## 2 Methods

### 2.1 Study subjects

This study adhered to the Declaration of Helsinki. This study obtained approval from the Ethics Committee of Meizhou People's Hospital/Meizhou Academy of Medical Sciences. Written informed consent forms were provided and signed by each participant. Full-time undergraduates who were undertaking a clinical internship and resident physicians training at the Meizhou People's Hospital between 2023 and 2024 academic year were selected as teaching subjects for this study. The inclusion criteria were as follows: 1) subjects who were currently studying for a bachelor's degree in clinical medicine and enrolled in an internship or resident physicians who were in the first year of standardized training in our institution; and 2) subjects who had completed a gynecology-related theoretical course and were in the primary stage of clinical practice. The exclusion criteria were as follows: 1) subjects with experience in gynecologic clinical practice; and 2) subjects unable to participate in all of the teaching sections.

### 2.2 Evaluation of the teaching effect

The theoretical assessment was performed with a closed book standardized examination paper in an anonymous way. The full score of the examination paper was 100 points with the content covering the following areas: ① the pathologic mechanism, anatomic structure, clinical manifestations, and the diagnostic points of gynecologic malignant tumors; ② identification of the invasive range of the tumor and the spatial relationship with surrounding tissues; and ③ formulation and preoperative evaluation of the general treatment plan. A 5-point Likert scale was adopted for the standardized questionnaire and each indicator was rated from 0 (no improvement) to 5 points (significant improvement). The students provided the rating based on the reality anonymously. All theoretical examination papers are uniformly numbered anonymously. The examination papers only indicate random numbers and do not contain students' names or group information. Teachers who did not participate in the implementation of this teaching are assigned to grade the papers based on the unified standard answers. The grading teachers are unaware of the group to which each paper belongs throughout the process, to avoid subjective scoring bias caused by knowing the grouping.

The self-designed standardized questionnaire (Supplemental file 2) was developed specifically for this study. The development process comprised three stages. First, the preliminary item pool was constructed based on a comprehensive review of commonly used medical education evaluation instruments, encompassing four core dimensions: subjective learning experience (learning interest, participation, self-learning, and teamwork), cognitive abilities (comprehensive and clinical thinking), visual-spatial perception (effectiveness of visual teaching aids), and clinical simulation scenarios (SP-based consultation and communication). Second, the initial draft was reviewed and refined by an expert panel consisting of three senior medical educators and two experienced gynecologic oncologists, who evaluated the content validity, relevance, and clarity of the items. Third, a pilot test was conducted with 20 residents (excluded from the final sample) to ensure the feasibility and comprehensibility of the questionnaire; minor revisions to the wording were made accordingly.

The student-based subjective evaluation included assessment of learning interest, course participation, self-learning capacity, comprehensive analytic capacity, clinical thinking capacity, teamwork capacity, effectiveness of visual teaching aids, and simulation of clinical consultation scenarios constructed using SP (simulated scenarios for clinical consultation, physical examination, and clinical doctor-patient communication). The teaching satisfaction evaluation included understanding of teaching content, course participation, improvement of learning motivation, and overall teaching satisfaction.

The unified Teaching Observation and Evaluation Scale was adopted for the evaluation of students by the same teaching team who gave the training courses. The Kappa value of the consistency assessment among the three evaluators was 0.825. The assessment was performed following the completion of the CBL + SP teaching stage, and the 3D model teaching. The students in the two groups were evaluated according to the following items: 1. active learning ability; 2. logical thinking ability; 3. course participation; 4. anatomic spatial cognition; 5. physician-patient communication ability; 6. team collaboration performance; and 7. clinical operation simulation skills.

### 2.3 Sample size calculation

G*Power software (version 3.1.9.6; company, Dusseldorf, Germany) was used to calculate the sample size for this study. The main outcome indicator of the study was student learning effectiveness. A statistical power of 80% and a 2-tailed ɑ = 0.05 were assumed. A sample size of 50 students per group was determined based on an effect size of 0.5 (Cohen's d) (13). A potential dropout rate of 10% was considered and a total of 110 students were recruited.

### 2.4 Teaching methods and study design

#### 2.4.1 Design of the study

The teaching subjects were numbered randomly by a nurse who was blinded to the study. The nurse divided the subjects into observation (CBL + SP + 3D) and control groups (CBL + SP) according to the random numbers assigned. For example, number 1 was assigned to the CBL + SP + 3D group, number 2 was assigned to the CBL + SP group, number 3 was assigned to the CBL + SP + 3D group, number 4 was assigned to the CBL + SP group, and so on. This grouping was completed until all numbers were assigned.

All teaching was completed by the same teaching team, which consisted of 3 physicians with ≥ 15 years of clinical experiences to assure the consistency and standardization of the teaching intervention. These three physicians underwent standardized training before teaching. A quantitative evaluation was performed on the students in both groups after training, including an objective assessment (standardized test paper) and self-designed standardized questionnaire.

#### 2.4.2 Teaching preliminary preparation

First, typical gynecologic malignant tumors, such as cervical, ovarian, and endometrial cancer, were selected according to the “Standardized training standards for resident physicians of China” and the actual clinical data were combined to form the teaching cases. Second, systemic training was performed on SPs, including the roles played (consultation, physical examination, and emotional response simulation), as well as evaluation and feedback. Third, Mimics 21.0 software (Materialise Co. Ltd., Leuven, Belgium) was used to establish the 3D model and identify and mark the tumor and adjacent structures, including the uterus, adnexa, pelvic blood vessels, nerves, rectum, and bladder, based on the MRI imaging data from the patients. Finally, a Form 3D printer (Formlabs Inc., Boston, USA) from an external institute was used to generate the solid color model.

#### 2.4.3 Teaching implementation

The three-dimensional integrated teaching mode was performed in three steps based on CBL + SP. First, the SP teaching method section was performed among all students in both groups. The students completed the consultation, physical examination, and clinical diagnosis, and the general treatment plan was formulated with the guidance of teachers on site. General and abdominal examination (inspection, palpation, percussion, and auscultation) was performed directly on the SPs to evaluate abdominal masses, ascites, and peritoneal irritation signs. Gynecologic pelvic examination (speculum examination and bimanual palpation of the cervix, uterus, and adnexa) was performed on high-fidelity pelvic simulators rather than on the SPs, given the invasive nature and ethical considerations of this procedure. The SPs were present during the simulator-based session and actively provided emotional and pain-related feedback (e.g., verbal responses or facial expressions) to simulate a real clinical encounter. All procedures were closely supervised by the teaching physicians to ensure correct techniques and student safety. Second, the CBL section discussion was performed among all students in both groups. The students were divided into groups with 4–5 students in each group to analyze the case, integrate the laboratory and imaging data to form the diagnosis and treatment plan, and several rounds of discussion were also performed. Finally, the 3D model teaching section was performed on the students in the CBL + SP + 3D group. MRI imaging data from real patients were used for the 3D reconstruction and modeling and 3D printing technology. Mimics 21.0 software was used for 3D modeling and the Form 3D printer was used to create a 3D solid model of a tumor. The 3D solid models and their specifications were illustrated in Supplemental file 1. The students used the 3D solid model to learn about tumor onset and the development space, the spatial localization, invasion pathway, clinical manifestations, and preoperative planning methods to improve the recognition of the space.

One week prior to the training, teachers provided students with a preview of the syllabus. Students independently reviewed the textbooks related to gynecologic malignant tumors (including cervical cancer, ovarian cancer, and endometrial cancer) and expanded their understanding accordingly. The training began with theoretical instruction, covering key topics such as the mechanism of gynecologic malignant tumors, the occurrence and development of the disease, its pathological classification and metastasis routes, clinical manifestations, diagnostic key points, and treatment principles. Following this, SP training was conducted. After SP training, CBL sessions were held. During this phase, traditional two-dimensional graphic teaching was utilized, with PowerPoint presentations outlining the structure of gynecological pelvic organs, disease progression, pathology, morbidity, clinical manifestations, differential diagnosis, and treatment. The two-dimensional interpretation of magnetic resonance images was also presented using PowerPoint. The total duration of the SP and CBL training was 120 min.

The 3D model and 3D imaging interpretation of the occurrence, development, clinical manifestations and treatment of gynecologic malignant tumors were conducted in the CBL + SP + 3D group. A 3D MRI model served as a visual aid, with Visari 3D Anatomy (Xi'an Visari Digital Technology Co., Ltd., Xi'an, China) and 3D Organon Anatomy software (Medis Media Pty Ltd, Queensland, Australia) supporting the process. The printed 3D model was then used to explain the lesion in details. The teacher guided the 3D model instruction, prompting questions and facilitating student responses. Students also engaged in group discussions to enhance their understanding. A schematic diagram (Figure 1) illustrates the sequence and timing of each component in the SB + CBL+3D model training.

**Figure 1:**
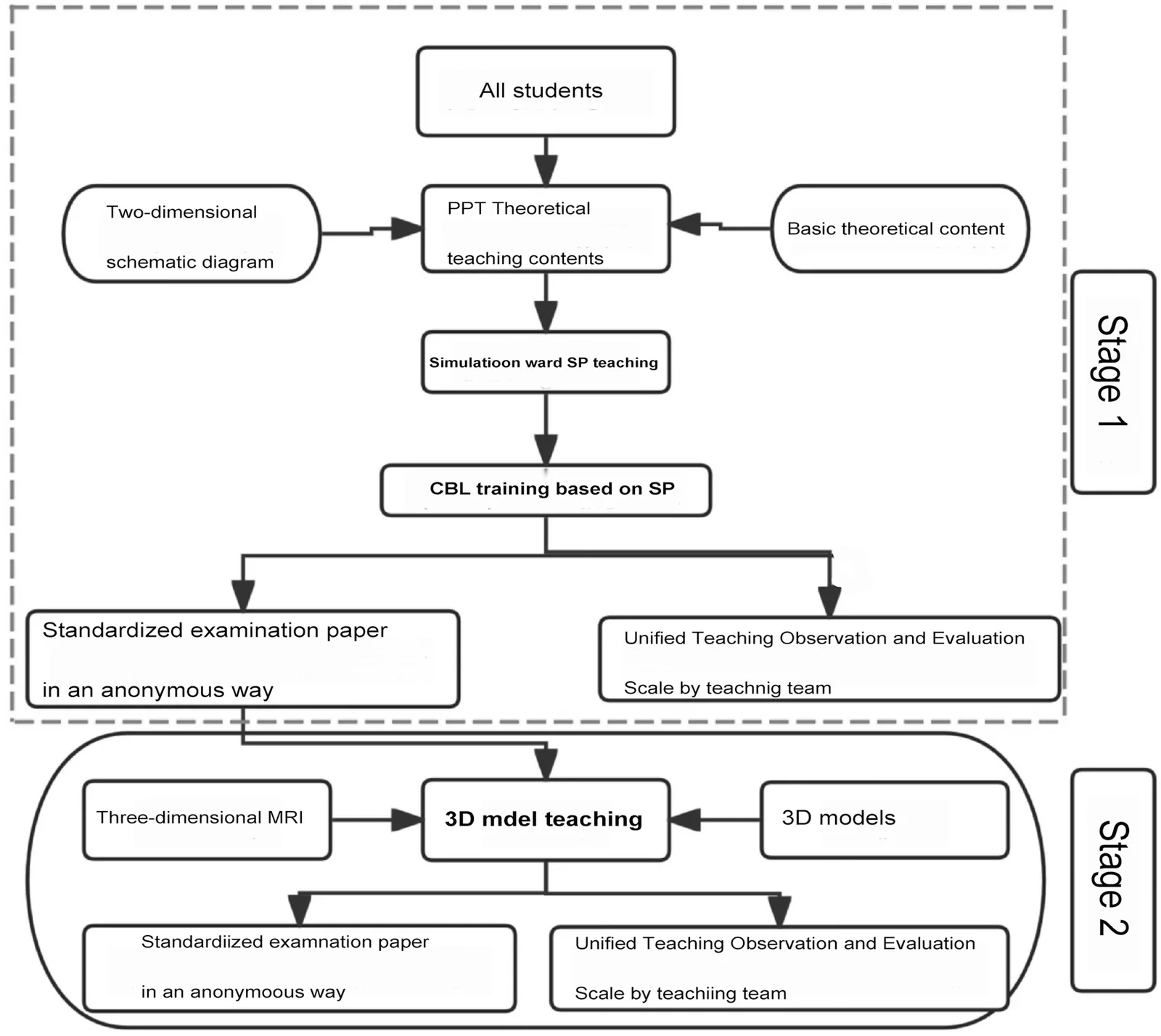
The flow chart of the SB + CBL+3D model training.

### 2.5 Statistical methods

All data were analyzed with SPSS 26.0 statistical software. The measurement data are expressed as the mean ± standard deviation (x ¯± s) and independent sample t-tests were used for inter-group comparisons. The counting data are expressed as *n* (%) and the *χ*² test was adopted. A *P* < 0.05 was considered to have statistical significance.

## 3 Results

A total of 110 students were enrolled in the study with 55 students in each group. One student in the CBL + SP + 3D group was interrupted from the internship due for a family issue and was unable to continue the training. One student in the CBL + SP group was transferred to another hospital from the internship unit and did not continue participation in this study. Therefore, 54 students were enrolled in each group.

### 3.1 Comparison of theoretical exam scores

The results of the closed book standardized theoretical examination showed that the CBL + SP + 3D group (83.60 ± 7.40) had a significantly higher average score than the CBL + SP group (75.90 ± 6.70; t = −5.66, *P* < 0.001, Table 1).

**Table 1:** Standardized theoretical examination scores between two groups (x¯ ± s).

Groups	Average scores	t	P
CBL + SP (*n* = 54)	76.07 ± 5.45	−7.69	<0.001
CBL + SP + 3D (*n* = 54)	83.93 ± 5.16		

### 3.2 Result of the students' subjective evaluation questionnaire and the formative assessment results

The results of the students' subjective evaluation questionnaire (Table 2, Figure 2A) demonstrated that the scores for the CBL + SP + 3D group were significantly higher than the CBL + SP group with respect to learning interest, classroom participation, self-learning capacity, comprehensive analytic capacity, clinical thinking capacity, effectiveness of visual teaching aids, and simulation of clinical consultation scenarios constructed using SP (*P* < 0.001). There was no significant difference in the teamwork capacity between the two groups (*P* = 0.154). The formative assessment results for the CBL + SP + 3D group was significantly higher than the CBL + SP group (*P* < 0.001).

**Table 2:** Result of the students' subjective evaluation questionnaire [Likert level 5 rating] (x¯ ± s).

Indicators	CBL + SP (*n* = 54)	CBL + SP + 3D (*n* = 54)	Z	*P*
Learning interest	4 (4,4)	5 (5,5)	−6.519	<0.001
Course participation	4 (3,4)	4 (4,5)	−4.20	<0.001
Self-learning capacity	4 (3,4)	4 (4,5)	−6.060	<0.001
Comprehensive analytic capacity	4 (3,4)	4 (4,5)	−5.222	<0.001
Clinical thinking capacity	4 (4,4)	4 (4,5)	−5.003	<0.001
Teamwork capacity	4 (4,4)	4 (4,5)	−3.714	0.154
Effectiveness of visual teaching aids	4 (3,4)	5 (4,5)	−7.065	<0.001
Simulation of clinical consultation scenarios constructed using SP	4 (3,4)	4.5 (4,5)	−6.320	<0.001
Formative scores	3 (3,3.6)	4.3 (4,4.8)	−8.61	<0.001

**Figure 2:**
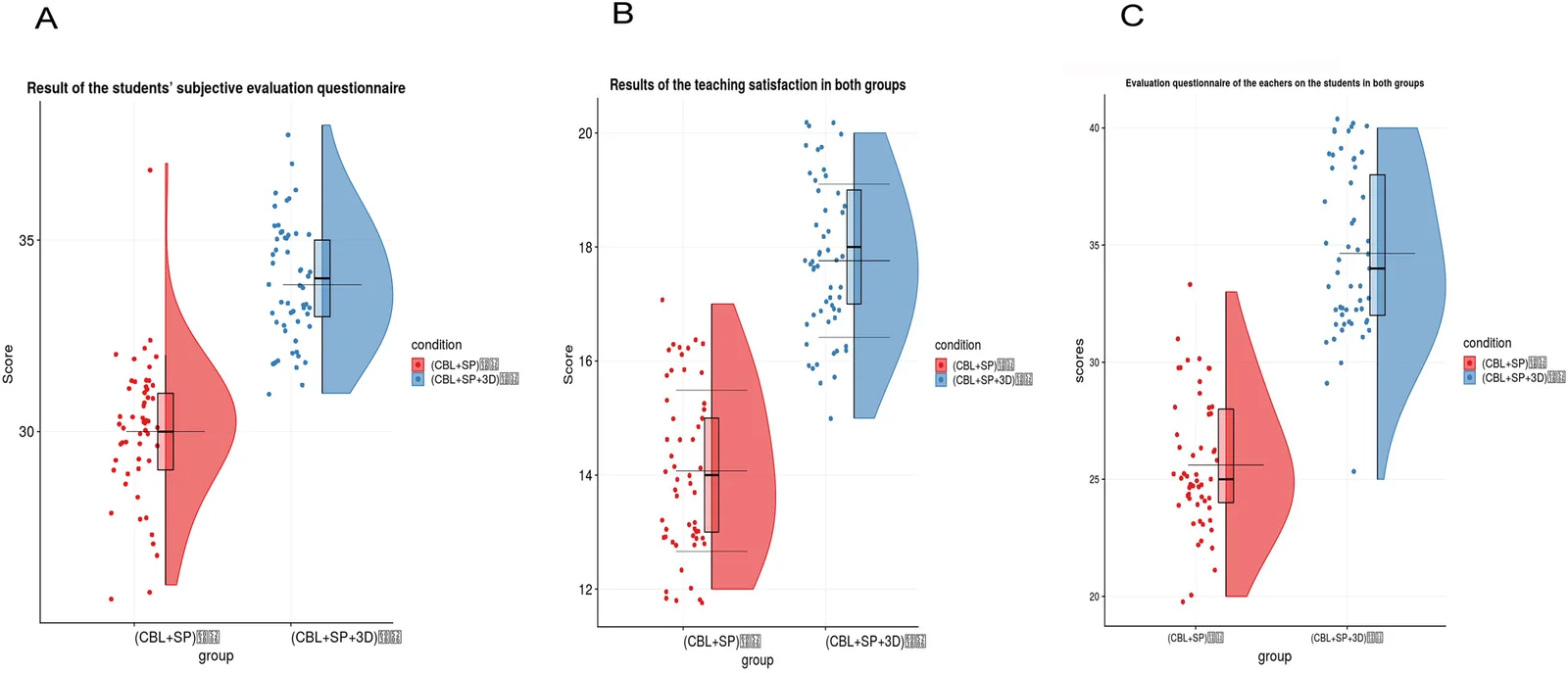
Violin plots of scores between the two groups. **(A)** Students' subjective evaluation scores; **(B)** the students satisfaction scores on teachers; **(C)** teacher evaluation scores on students' performance.

### 3.3 Results of the students' satisfaction questionnaire

The results of the teaching satisfaction (Table 3, Figure 2B) demonstrated that the CBL + SP + 3D group had significantly higher scores than the CBL + SP group in the multiple dimensions, including “understanding of the teaching content,” “course participation,” “improvement of the learning motivation,” and “overall satisfaction with teaching” (*P* < 0.001). The total score of satisfactions in the CBL + SP + 3D group was significantly higher than that in the CBL + SP group (44.63 ± 2.21 vs. 41.43 ± 2.27, t = −7.43, *P* < 0.001).

**Table 3:** Results of the teaching satisfaction in both groups (x¯ ± s).

Dimensions	CBL + SP (*n* = 54)	CBL + SP + 3D (*n* = 54)	Z	P
Understanding of the teaching content	3 (3,4)	5 (4,5)	−7.564	<0.001
Overall satisfaction with teaching	3 (3,4)	5 (4,5)	−6.935	<0.001
Improvement of the learning motivation	4 (3,4)	4 (4,5)	−6.060	<0.001
Course participation	3 (3,4)	4 (4,5)	−6.199	<0.001

### 3.4 Results of the teacher evaluation questionnaire

The results of the evaluation questionnaire by the 5 teachers for the students in both groups are shown in Table 4 and Figure 2C. The students in CBL + SP + 3D group had higher scores than the CBL + SP group in the following indicators: active learning; clarity of diagnosis and treatment logic; course participation; anatomic spatial cognition; physician-patient communication skills; teamwork performance; and clinical operation simulation skills (*P* < 0.001). The total scores by the teachers in the CBL + SP + 3D group was significantly higher than that in the CBL + SP group (34.65 ± 3.59 vs. 25.61 ± 2.88, t = −14.44, *P* < 0.001).

**Table 4:** Evaluation questionnaire of the 5 teachers on the students in both groups (x¯ ± s).

Indicators	CBL + SP (*n* = 54)	CBL + SP + 3D (*n* = 54)	t	P
Active learning	3 (2,3)	4 (4,4)	−7.76	<0.001
Clarity of diagnosis and treatment logic	3 (3,4)	4 (4,5)	−7.43	<0.001
Course participation	3 (3,4)	4 (4,5)	−7.86	<0.001
Anatomic spatial cognition	3 (2,3)	4 (4,4)	−7.76	<0.001
Physician-patient communication skills	3 (2,3)	4 (4,5)	−9.03	<0.001
Teamwork performance	3 (3,3)	4 (4,5)	−7.69	<0.001
Clinical operation simulation skills	3 (3,4)	4 (4,5)	−7.33	<0.001

## 4 Discussion

In this study the CBL + SP + 3D group was shown to have significantly higher scores than the CBL + SP group on the following items: average theoretical exam; the students' subjective evaluation questionnaire and the formative assessment results, students' satisfaction evaluation and the teachers' evaluations on the course interventions. The results of this study were consistent with other medical related teaching study results (6–9), indicating that 3D technology significantly improved the effect of clinical teaching in the medical department and the learning efficiency of the medical students, which warrants further promotion.

The present study was undertaken in response to the inherent complexity of pelvic anatomy in gynecologic oncology, wherein tumor infiltration, vascular encasement, and metastatic pathways exhibit intricate three-dimensional relationships that are inadequately conveyed through conventional two-dimensional imaging and lecture-based instruction. Although CBL and SP methodologies effectively cultivate clinical reasoning and communication competencies, neither modality addresses the fundamental deficit in stereoscopic anatomical cognition. Furthermore, 3D printing's integration with CBL and SP in gynecologic oncology education remains underexplored. Accordingly, the pedagogical rationale for incorporating 3D technology is threefold: (i) to transform two-dimensional MRI data into tangible, manipulable models that elucidate spatial relationships between tumors and adjacent pelvic structures; (ii) to simulate tumor invasion boundaries and resection margins, thereby facilitating preoperative surgical planning; and (iii) to shift the learning paradigm from passive knowledge acquisition to active stereoscopic reasoning, thereby reinforcing comprehensive clinical decision-making.

In this study a ternary integrated teaching mode was established, which systematically integrated SPs, CBL, and 3D reconstruction printing technology based on cognitive load and constructivist learning theory, which integrate knowledge acquisition, scenario shifting, and skill construction to form a multi-level, end-to-end teaching loop. Innovation of this system is demonstrated in the following three aspects: First, virtuality was combined with the physical model, breaking through the limitations of time and space, making abstract pathology concrete, and helping students intuitively understand tumor infiltration and the three-dimensional structure. The 3D printed model enhanced the perception of tumor texture and boundaries through tactile feedback, which significantly improved spatial cognition (14). The majority of the students in the observation group indicated that the 3D model helped them better understand the occurrence and development of tumors, vascular wrapping, resection boundaries, and surgical difficulties. 3D technology enables students to become more familiar with anatomic structures, stimulating their learning interest and subjective initiative, and reflecting the transformation of teaching from knowledge imparting to cognitive construction, which significantly improved spatial cognitive ability (6, 7). Second, the 3D model operations were integrated into SP scenarios, simulating patient privacy, symptoms, and emotions, promoting student understanding of the social and psychological background of the disease, and enhancing the physician-patient communication and clinical thinking abilities (6, 7, 10). Third, the evaluation system emphasizes comprehensive analysis ability. For example, the evaluation showed the hidden lesions through 3D models and facilitated forming advanced thinking from the phenomenon of cognition to pathologic analysis and diagnosis and treatment decision-making under the guidance of teachers, strengthening the knowledge system, logical reasoning, team collaboration, and enhancing clinical decision-making ability. The results of this study showed that students in the CBL + SP + 3D group performed significantly better on multiple indicators than the CBL + SP group, including the content comprehension, model-assisted teaching, clinical thinking, comprehensive analysis, verifying the advantages of the mode in regulating cognitive load, improving learning outcomes, and profound understanding of clinical structures and diagnostic strategies. However, there are very few studies like this study, making it impossible to perform horizontal comparisons.

els; (ii) CBL sessions incorporating physical 3D models for case analysis; (iii) SP simulation with model-based correlation of physical findings to anatomical pathology-3; (iv) preoperative planning and surgical debriefing using 3D models; and (v) formative assessment incorporating 3D model-based anatomical identification and tumor invasion evaluation. Regarding cost-related concerns, mitigation strategies include batch reuse of printed models across multiple trainee cohorts, cloud-based archiving of digital reconstructions for on-demand access, progressive integration of VR/AR technologies to reduce dependency on physical models, and allocation of institutional educational funding to preclude financial burden on trainees. These strategies collectively support the sustainable implementation of 3D technology in routine residency curricula.

Although the teaching mode applied to the CBL + SP + 3D group in this study achieved active progress, there were still many limitations. First, this was a single-center study which requires verification from a multi-center study. Second, the equipment for the 3D reconstruction and printing technology still cost relatively high, and the reservation of the teacher resources with the related technology is still limited, resulting in obstacles promoting the results of this study. Combining digital anatomy and virtual simulation technologies, such as virtual reality/ augmented reality, to reduce reliance on physical models, establishing a teacher competency development plan to enhance the integration of teaching and technology are recommended. Third, the evaluation system used in this study involved a self-designed assessment questionnaire. Our self-designed student questionnaire was not statistically validated prior to the study, which may limit the generalizability of the subjective findings, and may lead to bias of the results. Reforming the assessment system and introducing multiple mechanisms, such as the process evaluation, objective structured clinical exam (OSCE), and SP feedback are recommended to reflect teaching effectiveness in a more comprehensive way. Fourth, the evaluations on the students and teachers training were not conducted in a blinded manner, as they relied on classroom performance for scoring. This may result in potential bias on the evaluation results. Introducing blinding evaluation scales might be necessary for upcoming research to prevent bias.

The 5G network and AI technology can be considered to realize the remote real-time surgical observation in a corollary study, allowing the teachers to use the 3D technology for teaching and guiding the students in observing the details of tumor resection. The 5G network and AI technology can also be combined with intelligent assistance systems to analyze the surgical risks and provide personalized teaching plans, which can also be taught remotely, allowing students from other centers to observe the teaching and surgery remotely in real time.

## 5 Conclusion

This study demonstrated that introducing 3D reconstruction and printing technology under the CBL + SP teaching framework as the intuitive teaching tools can effectively enhance students' stereoscopic cognition of the complex anatomy of gynecologic tumors and pathologic structures, and significantly facilitate improvement of students' clinical thinking and comprehensive analysis ability. However, multiple verifications from the multicenter and structured evaluations are still needed for these results.

## Data Availability

The raw data supporting the conclusions of this article will be made available by the authors, without undue reservation.

## Supplementary material

Supplemental File 1 Photographs of the 3D models and the model specifications.

Supplemental File 2 English copy of the questionnaire.

## References

[1] Cibula D Raspollini MR Planchamp F Centeno C Chargari C Felix A. ESGO/ESTRO/ESP guidelines for the management of patients with cervical cancer - update 2023. Int J Gynecol Cancer. (2023) 33(5):649–66. 10.1136/ijgc-2023-004429 37127326 PMC10176411

[2] Zhu B Gu H Mao Z Beeraka NM Zhao X Anand MP. Global burden of gynaecological cancers in 2022 and projections to 2050. J Glob Health. (2024) 14:04155. 10.7189/jogh.14.04155 39148469 PMC11327849

[3] Recker F Haverkamp N Mustea A Gembruch U Raupach T. Application of test-enhanced learning (TEL) in obstetrics and gynecology: a prospective study. Arch Gynecol Obstet. (2022) 306(5):1563–71. 10.1007/s00404-022-06656-4 35831757 PMC9281379

[4] Sabrina F Ilkka K Mervi HN Charles R Simon L Ameli T. Fostering prevention of cervical cancer by a correct diagnosis of precursors: a structured case-based colposcopy course in Finland, Norway and UK. Cancers (Basel). (2020) 12(11):3201. 10.3390/cancers12113201 33143157 PMC7692698

[5] Wilbur K Elmubark A Shabana S. Systematic review of standardized patient use in continuing medical education. J Contin Educ Health Prof. (2018) 38(1):3–10. 10.1097/CEH.0000000000000190 29517612

[6] Zhao J Gong X Ding J Xiong K Zhuang K Huang R. Integration of case-based learning and three-dimensional printing for tetralogy of fallot instruction in clinical medical undergraduates: a randomized controlled trial. BMC Med Educ. (2024) 24(1):571. 10.1186/s12909-024-05583-z 38789956 PMC11127445

[7] Feng S Sun YJ Zhu QR Shi SF Zhang YS Yuan F. Integration of 3D printing and case-based learning in clinical practice for the treatment of developmental dysplasia of the hip. BMC Med Educ. (2024) 24(1):986. 10.1186/s12909-024-05934-w 39256737 PMC11389423

[8] Yan X Zhu Y Fang L Ding P Fang S Zhou J. Enhancing medical education in respiratory diseases: efficacy of a 3D printing, problem-based, and case-based learning approach. BMC Med Educ. (2023) 23(1):512. 10.1186/s12909-023-04508-6 37461009 PMC10353117

[9] Zhang W Chen Y Huang X. The application of CT 3D reconstruction and 3D printing technology combined with CBL teaching mode in the clinical teaching of joint orthopedics. Adv Med Educ Pract. (2025) 16:535–43. 10.2147/AMEP.S509201 40201411 PMC11977560

[10] Wang T Wen Y Wang Z. nnU-Net based segmentation and 3D reconstruction of uterine fibroids with MRI images for HIFU surgery planning. BMC Med Imaging. (2024) 24(1):233. 10.1186/s12880-024-01385-3 39243001 PMC11380377

[11] Mercorio A Zizolfi B Barbuto S Danzi R Di Spiezio Sardo A Moawad G. Three-dimensional imaging reconstruction and laparoscopic robotic surgery: a winning combination for a complex case of multiple myomectomy. Fertil Steril. (2023) 120(1):202–4. 10.1016/j.fertnstert.2023.04.015 37085096

[12] Huerta-Reyes M Aguilar-Rojas A. Three dimensional models to study breast cancer (review). Int J Oncol. (2021) 58(3):331–43. 10.3892/ijo.2021.5176 33650654

[13] Zhao W He L Deng W Zhu J Su A Zhang Y. The effectiveness of the combined problem-based learning (PBL) and case-based learning (CBL) teaching method in the clinical practical teaching of thyroid disease. BMC Med Educ. (2020) 20(1):381. 10.1186/s12909-020-02306-y 33092583 PMC7583209

[14] Fan KS Durnea C Nygaard CC Khalil M Doumouchtsis SK. Three-dimensional volume rendering of pelvic floor anatomy with focus on fibroids in relation to the lower urogenital tract based on cross-sectional MRI images. J Med Syst. (2023) 47(1):62. 10.1007/s10916-023-01947-y 37171621 PMC10181971

