# Supplementary material for: Application of 3D reconstruction combined with 3D printing and CBL + SP in teaching gynecologic malignant tumors
Source: Front Med (Lausanne). 2026 Sep 14;13:1915577. doi: 10.3389/fmed.2026.1915577 (PMC13617124; doi:10.3389/fmed.2026.1915577)
Supplement: Supplementary file 1 [file Table1.docx]

Supplemental file 1. photographs of the 3D models and the model specifications

| Categories | Specification |
| --- | --- |
| Model coverage structure | 1. Core organs: Cervix (including the cervical-vaginal part and cervical canal), lower segment of the uterine body, upper segment of the vagina (2-3cm); 2. Key supporting tissues: main ligament, sacral ligament, vesicular-cervical ligament, rectovaginal septum; 3. Metastasis-related structures: Pelvic lymph nodes, para-aortic lymph nodes; 4. Tumor-related structures: Primary cervical tumor, Lesions of parauterine invasion, vaginal invasive lesion, metastatic lymph nodes   All parts were detachable |
| Size and proportion | 1. Proportion：1:2  2. Overall size：Length: 22-28cm, width:16-20cm; height:20-24cm； |
| Materials | 1. Cervical body: PLA eco-friendly resin (Shore D 65-70) in light pink, tough in texture, completely and realistically presenting the texture of the cervical muscle layer;  2. Normal cervical mucosa: Flexible resin (Shore A 50-55) in light red with a thickness of 0.8-1mm, adhering to the surface of the cervix;  3. Cervical cancer tissue: Flexible resin (Shore A 40-45) iin deep red, soft and elastic to the touch, forming a strong visual and tactile distinction from normal cervical tissues;  4. Parauterine ligament (main ligament/sacral ligament) : Flexible resin (Shore A 35-40) in milky white,being slightly bendable and simulating the elasticity and toughness of the ligament;  5. Upper vaginal segment: Semi-transparent PLA resin in light pink, facilitating the observation of the depth of vaginal invasion by the tumor;  6. Pelvic lymph nodes: Hard resin (Shore D 50), with normal lymph nodes in white, and metastatic lymph nodes in deep red (the color is consistent with the material of the cervical cancer tissue). |
| Detailed precision | 1. Basic parameters of organs are as follows (Error≤0.4mm). Diameter of the cervical canal: 2.5mm，Cervical and vaginal length: 2.8cm，Muscle layer thickness: 3.5mm. Width of the main ligament: 1.2cm, The width of the sacral ligament: 1cm. The attachment points are precise;  2. Core parameters of tumors：   1. Primary lesions: there are two types, exophytic type and ulcerative type. exophytic type looks like cauliflower, with a diameter of 4cm, and the surface protrusion spacing 0.3-0.5cm; Ulcerative type is set to have a diameter of 3.5cm and a central depression of 0.8cm. The default setting is exophytic type, which is the most common type. 2. Range of invasion: Parauterine invasion length at 1cm (stage IIB standard), vaginal invasion depth at 1.5cm (stage IA1 standard), with the error ≤0.5mm.   3. Lymph node parameters: Diameter is set to 0.9cm, fixed arrangement according to anatomical position |
| Structural design | 1. Detachable parts：  Sagittal plane dissection of the cervix (2 lobes), exposing the tumor in the cervical canal and myometrial infiltration; The parauterine ligaments can be removed separately (one on each side)； The upper segment of the vagina can be split (1 segment). Lymph nodes are disassembled in 4 groups, with 2 parts in each group;  2. The base cannot be disassembled, and use an anti-slip rubber base of 15cm×12cm. |
| Printing technology | 1. The layer height is set at 0.08mm to balance printing accuracy and printing efficiency.  2. Filling density: 30% for the cervical body, 35% for tumor tissue, 25% for ligaments, 20% for lymph nodes;  3. Support structure is set to be tree-shaped, with a contact area ≤4mm²;  4. Post-treatment: Surface roughness Ra≤0.8μm, no burrs, split surface gap ≤0.3mm, tumor surface retains its natural shape. |
