# Supplementary material for: Application of 3D reconstruction combined with 3D printing and CBL + SP in teaching gynecologic malignant tumors
Source: Front Med (Lausanne). 2026 Sep 14;13:1915577. doi: 10.3389/fmed.2026.1915577 (PMC13617124; doi:10.3389/fmed.2026.1915577)
Supplement: Supplementary file 2 [file Table2.docx]

1. Students’ Questionnaire

This questionnaire is filled anonymously, aiming to understand your subjective feeling and feedback of the current teaching mode. All of the data were for the purpose of scientific research only.

| Serial No. | Items for evaluation | Completely no | Slightly improved | Moderately improved | Significant improved | Greatly improved | |
| --- | --- | --- | --- | --- | --- | --- | --- |
| 1 | Learning Interest | ☐ | ☐ | ☐ | ☐ | ☐ |  |
| 2 | Self-learning capacity | ☐ | ☐ | ☐ | ☐ | ☐ |  |
| 3 | Comprehensive analytical capacity | ☐ | ☐ | ☐ | ☐ | ☐ |  |
| 4 | Clinical thinking capacity | ☐ | ☐ | ☐ | ☐ | ☐ |  |
| 5 | Teamwork capacity | ☐ | ☐ | ☐ | ☐ | ☐ |  |
| 6 | Understanding of teaching content | ☐ | ☐ | ☐ | ☐ | ☐ |  |
| 7 | classroom participation activity | ☐ | ☐ | ☐ | ☐ | ☐ |  |
| 8 | Effect of the learning assisted by visual teaching aids | ☐ | ☐ | ☐ | ☐ | ☐ |  |
| 9 | Simulation of clinical consultation scenarios constructed using SP | ☐ | ☐ | ☐ | ☐ | ☐ |  |
| 10 | Overall satisfaction of the teaching | ☐ | ☐ | ☐ | ☐ | ☐ |  |

Please write down any other suggestions or comments, if any(optional):

2．【Manual of the scoring rules for the teachers(Teaching Observation and Evaluation Scale)】

for the classroom performance scoring of the combined teaching mode of CBL+SP+3D and the 5-grade scoring criteria is used.

| Items | Explanation of the indicators | Score range |
| --- | --- | --- |
| 1. Classroom participation of the students | Whether the student is active for the speech, questions asking, participation of the discussion and demonstration | 1–5 |
| 2. The logical thinking capacity of the students | Whether the process of the clinical reasoning is clear and whether there is any structured expression | 1–5 |
| 3. Anatomical spatial cognition | The accuracy of the recognition of the structure within if 3D model and the clearance of the spatial description | 1–5 |
| 4. Doctor-patients communication capacity | Whether the communication skills, attitude and the phrasing are compliance with the standards during the SP interaction | 1–5 |
| 5. Teamwork performance | Whether the student is active for the teamwork and whether the task assignment is balanced | 1–5 |
| 6. The stimulation skills of the clinical operation | Whether the stimulation of the process of examination and treatment are compliance with the standards and whether they are proficient | 1–5 |
| 7. Active learning capacity | Whether the student have full pre-class preparation and in-depth data search and after-class active learning extension | 1–5 |

Each item has a maximum score of 5 points with the total score of 40 points (5=outstanding performance, 1=completely failed to meet the standard).

3．The formative assessment scale of the students
This scale is for the evaluation of the students’ classroom performance and capacity development during the CBL+SP+3D printing teaching process. The Likert 5-level scoring method is used(1=underperformance, 5=outstanding performance).

| Dimension | Specific Items | Rating Scales（1~5 points） | Weight（%） | Remarks | Score |
| --- | --- | --- | --- | --- | --- |
| 1. Classroom participation and the initiative | - Attendance rate - Frequency of speech in the classroom - Participation of the group discussion | 1–5 | 20% | The classroom records by the teachers are required |  |
| 2. Self-learning and the pre-class preparation | - The completion of the the case preview - The data cited during the lesson - The completion of the demonstration contents | 1–5 | 20% | The pre-class task lists should be combined |  |
| 3. The clinical thinking and the logic of the diagnose and treatment | - The analytical capacity of the disease - The logicality of the diagnose process - The design of the treatment plan | 1–5 | 25% | The score should be based on the classroom performance and the group demonstration |  |
| 4. Application of the model and the spatial recognition | - Model recognition capacity - Accuracy of the structure positioning - Spatial planning capacity | 1–5 | 20% | The score should be based on the observation of the model operation process |  |
| 5. Communication, collaboration and humanistic care | - The communication skills with SP - Awareness of the group collaboration - Summary and the feedback capacity | 1–5 | 15% | Teacher observation+SP feedback |  |

Calculation formula of the total score：
Total score =（Score of the the classroom participation × 20%）+（Score of the self-learning × 20%）+（Score of the clinical thinking × 25%）+（Score of the spatial recognition × 20%）+（Score of the communication collaboration × 15%）

The explanation of the scoring criteria（Likert 5-level scoring）

| Grade | Score | Description |
| --- | --- | --- |
| 1 point | The performance was significantly insufficient and the student basically did not participate in such activity. |  |
| 2 points | The performance was insufficient and the student had low rate of participation which requires improvement. |  |
| 3 points | The student had average performance, reaching the minimum standard. |  |
| 4 points | The student performed well and completed the task with strong initiative. |  |
| 5 points | The student performed excellent with activity and initiative and fully completed the goal. |  |
